# Monocyte activation and cytokine production in Malawian children presenting with *P. falciparum* malaria

**DOI:** 10.1111/pim.12319

**Published:** 2016-04-28

**Authors:** W. L. Mandala, C. L. Msefula, E. N. Gondwe, M. T. Drayson, M. E. Molyneux, C. A. MacLennan

**Affiliations:** ^1^ Malawi‐Liverpool Wellcome Trust Clinical Research ProgrammeBlantyreMalawi; ^2^ Department of Biomedical SciencesCollege of MedicineUniversity of MalawiBlantyreMalawi; ^3^ Liverpool School of Tropical MedicinePembroke PlaceUniversity of LiverpoolLiverpoolUK; ^4^ Medical Research Council Centre for Immune Regulation and Clinical Immunology ServiceInstitute of Biomedical ResearchSchool of Immunity and InfectionCollege of Medicine and Dental SciencesUniversity of BirminghamBirminghamUK; ^5^ Department of MedicineCollege of MedicineUniversity of MalawiBlantyreMalawi; ^6^ The Jenner InstituteNuffield Department of MedicineUniversity of OxfordOxfordUK

**Keywords:** integrins, monocytes, toll‐like receptors and cytokines

## Abstract

Malaria in malaria‐naïve adults is associated with an inflammatory response characterized by expression of specific activation markers on innate immune cells. Here, we investigate activation and adhesion marker expression, and cytokine production in monocytes from children presenting with cerebral malaria (CM,* n* = 36), severe malarial anaemia (SMA,* n* = 42) or uncomplicated malaria (UM,* n* = 66), and healthy aparasitemic children (*n* = 52) in Blantyre, Malawi. In all malaria groups, but particularly in the two severe malaria groups, monocyte expression of CD11b, CD11c, CD18, HLA‐DR and CD86, and percentages of TNF‐α‐ and IL‐6‐producing monocytes were lower than in healthy controls, while expression of CD11a, TLR2 and TLR4 was lower in children with severe malaria compared with controls. These levels mostly normalized during convalescence, but percentages of cytokine‐producing monocytes remained suppressed in children with SMA. In all malaria groups, especially the SMA group, a greater proportion of monocytes were loaded with haemozoin than among controls. In a *P. falciparum* hyperendemic area, monocytes in children with acute symptomatic malaria have reduced expression of adhesion molecules and activation markers and reduced inflammatory cytokine production. This immune suppression could be due to accumulation of haemozoin and/or previous exposure to *P. falciparum*.

## Introduction

The acquisition of protective immunity against malaria requires repeated infections over a number of years 1. Particularly, during the early stages of a malaria infection, innate immunity, involving monocytes and other immune cells, plays a crucial role in controlling parasite growth rate 2. Monocytes are precursors of macrophages and dendritic cells. Their main functions are phagocytosis, antigen presentation and cytokine production 3. Pro‐inflammatory cytokines, such as TNF‐α and IL‐6 which are produced by monocytes among other cells, are elevated in severe malaria and appear to be important for controlling infection 4. However, their excessive production has been linked to immunopathogenesis in malaria 5, 6.

Also implicated in immunity and pathogenesis of malaria are the adhesion molecules which are particularly important in the sequestration of parasitized red blood cells to vascular endothelium 7. During inflammation, upregulation of adhesion molecule expression serves to enhance cell‐to‐cell interactions and transmission of signals that direct effector functions. The major adhesion molecule families involved in leucocyte trafficking, activation and differentiation are the integrins, selectins (L‐selectin, E‐selectin, and P‐selectin) and immunoglobulin super‐family members 8.

The integrins are a large family of αβ‐heterodimers. Integrins incorporating β2 (CD18) are restricted to leucocytes and all are expressed on monocytes. CD11a, b and c all combine with CD18 to form different integrins. They bind a variety of ligands including ICAM‐1 and ICAM‐2 which are expressed by endothelial cells. Endothelial ICAM‐1 is a key factor in cytoadherence of malaria‐parasitized red blood cells 9.

HLA‐DR and CD86 are expressed on monocytes and serve as activation markers. While HLA‐DR is critical for cognate interactions associated with peptide presentation to αβ‐CD4^+^ T cells, CD86 is key for accessory signalling 10. Monocytes also express Toll‐like receptors (TLRs) which are pattern‐recognition receptors (PRRs) that recognize pathogen‐associated molecular patterns (PAMPs) including microbial pathogens, virulence factors and intracellular protozoan parasites 11. TLRs are involved in the initial activation of the innate immune system 12 and are capable of recognizing a wide range of microbial components including bacterial lipopolysaccharides (LPS), lipopeptides, and glycolipids, unmethylated bacterial DNA and viral nucleic acids 11.

*P. falciparum*‐derived glycosylphosphatidylinositol (GPI) is known to induce potent TNF‐α responses in macrophages 13. Recognition of these membrane components from *P. falciparum* is mediated by TLR‐2, and to a lesser extent, TLR‐4 14 and microparticles produced by infected red blood cells are also known to induce immune responses through the recognition of TLR‐4 15. TLR‐9, expressed particularly in either dendritic cells or B cells, but also at low levels on monocytes, T cells and NK cells 16, recognizes *P. falciparum* haemozoin 17, with DNA bound to proteins or nucleosomes being the true parasite ligand for TLR‐9 16, 18, 19, 20. TLR and other immune signalling pathways may be subject to downregulation following stimulation. Before the discovery of TLRs, it was known that stimulation with endotoxin (lipopolysaccharide, LPS), now known to be the major ligand of TLR‐4, induces tolerance to restimulation, both *in vitro* and *in vivo* 21.

During the intraerythrocytic life cycle of *P. falciparum*, haemoglobin is degraded but the parasite is unable to catabolize haem, which aggregates forming an insoluble polymer called malarial pigment or haemozoin 22. Neutrophils, monocytes and resident macrophages ingest haemozoin and haemozoin‐containing parasitized erythrocytes. Phagocytosis of haemozoin by monocytes has been shown to have both stimulatory effects, including induction of pro‐inflammatory cytokines 23, and inhibitory effects, including reduced phagocytosis and oxidative burst 24, and reduced expression of CD11c 25.

We conducted this study to explore expression of CD11a, CD11b, CD11c, CD18, HLA‐DR, CD86, TLR‐2 and TLR‐4 and production of TNF‐α and IL‐6 by monocytes in Malawian children presenting with different clinical forms of malaria.

## Materials and Methods

### Study population

For the first study, participants were children admitted with malaria to Queen Elizabeth Central Hospital (QECH), and medically well children attending surgical outpatient clinics at QECH and Beit Cure International Hospital, both in Blantyre. Demographic and clinical features of the participants have been reported before 26. In brief, children were enrolled during the rainy season (November 2005 to April 2006) after obtaining informed consent from the parent or guardian. Each child was examined by a research nurse and/or clinical officer, baseline demographic data were recorded, and a peripheral blood sample was collected.

The children were assessed for level of consciousness using the Blantyre Coma Score (BCS) on admission and during intensive clinical care. Over forty children were prospectively enrolled into each of the four clinical groups defined by diagnoses of CM, SMA, UM or healthy controls (Table S1). Malaria was defined as a clinical syndrome without an apparent alternative cause, in the presence of *P. falciparum* asexual parasites on blood film microscopy.

Children presenting with CM had a BCS of 2/5 or less at admission and 4 h later, while children in all other groups had a score of 5/5 at both times. Those presenting with SMA had a blood haemoglobin concentration of 5 g/dL or less, and all other children had a haemoglobin concentration above this level. Children who tested positive for HIV infection were excluded from the study and were referred to the antiretroviral therapy clinic.

Expression of surface markers on peripheral blood monocytes and proportion of haemozoin‐loaded monocytes were investigated. Following exclusions, 113 children with malaria (54 with UM, 30 with SMA, 29 with CM) and 42 healthy controls were recruited. Of the children with malaria, 73 (34 UM, 21 SMA and 18 CM) were successfully followed up a month after treatment.

For the second study, a further set of 41 children were recruited from November 2006 to February 2007 from the same health centres as the first set (Table S2) and using the same criteria: 12 with UM, 12 with SMA, 7 with CM and 10 healthy controls. Percentages of TNF‐α‐ and IL‐6‐producing monocytes were determined. Fourteen of thirty‐one (45%) children with malaria (6 UM, 4 SMA and 4 CM) were aparasitaemic when followed up a month after treatment. Convalescence data were only collected from well and aparasitaemic children.

### Ethics review and approval

Ethical approval for the study was obtained from College of Medicine Research and Ethics Committee (COMREC) and Ethics Committee of the Liverpool School of Tropical Medicine, UK. Written informed consent was obtained from the parent or guardian of every child before the child was recruited into the study. A 5‐mL venous blood sample was taken at the time of recruitment and in convalescence. Blood was collected in EDTA anticoagulant for immunophenotyping and heparin for cytokine production.

### HIV, malaria parasite and haemozoin tests

HIV testing was performed using two rapid tests; Determine HIV1/HIV2 (Abbott Laboratories, Japan) and Unigold (Trinity Biotech, Dublin), according to the manufacturers’ instructions. Malaria parasitaemia was determined by thick and thin blood films. Reading of the slides for malaria parasites and for haemozoin (performed for the first group only) was according to standard procedures 27.

### Immunophenotyping

For each sample, 25 μL of EDTA blood was mixed with 1 μL of three directly conjugated antibodies: a FITC‐conjugated antibody (anti‐CD11a, anti‐CD18, anti‐HLA‐DR, anti‐CD86), a PE‐conjugated antibody (anti‐CD11b, anti‐CD11c, anti‐TLR‐2, anti‐TLR‐4) and anti‐CD14‐APC. Samples were incubated for 15 min in the dark at room temperature. About 500 μL of 1× FACS lysing solution (Becton Dickinson, Franklin Lakes, NJ, USA) was added to each tube and incubated in the dark for 10 min at room temperature. Cells were washed twice with 2 mL of PBS and fixed with 100 μL PBS/1% formaldehyde. Data were acquired on a BD FACSCalibur flow cytometer and analysed using cellquest pro software (San Jose, CA, USA) as shown in Figure 4.

### Detection of TNF‐α‐ and IL‐6‐producing monocytes by flow cytometry

An aliquot of 1 mL of heparin blood was mixed with 10 μL of 100 μg/mL LPS and 10 μL of 1 μg/mL Brefeldin A (BFA). The mixture was vortexed and incubated at 37°C, 5% CO_2_ for 4 h in loose‐capped tubes. A negative control, containing 1 mL of blood from the same patient but without any LPS added, was also vortexed and incubated under the same condition. An aliquot of 50 μL of stimulated and unstimulated blood was labelled with 2 μL of anti‐CD14‐APC and incubated in the dark at room temperature for 15 min. 2 mL of 1× FACS lysis solution was added to each tube, vortexed and incubated in the dark for 10 min. Tubes were centrifuged at 1600 rpm and 4°C for 5 min, the supernatant aspirated and 500 μL of 1× FACS Perm (Becton Dickinson) added to each tube which was then incubated in the dark for 10 min. Cells were then washed with 2 mL of PBS/0·5% bovine serum albumin (BSA). Four microlitres of PE‐conjugated anti‐TNF‐α or anti‐IL‐6 was added to each tube. Each tube was vortexed and incubated for 30 min in the dark at room temperature, washed with 2 mL of PBS/0·5% BSA and the cells fixed with 200 μL PBS/1% formaldehyde. Data were acquired and analysed on a FACSCalibur instrument within an hour. We monitored the calibration of our flow cytometer a minimum of once per week using calibrite beads and fascomp software (both Becton Dickinson).

**Figure 1:**
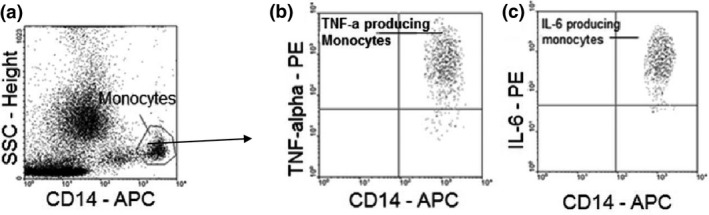
Gating strategy for determining the monocyte population in the side‐scatter/CD14 plot (a) the proportion of TNF‐alpha‐producing (b) and IL‐6‐producing (c) monocytes.

Data were analysed using cell quest software (Becton Dickinson). The gating procedure was conducted as shown in Fig. 1. Samples were gated on monocytes by side scattered light and CD14 expression. Results were expressed as the percentage of cytokine‐producing cells in the CD14+ cell population. The following formula was used for calculating the percentage of cytokine‐producing monocytes:

$$\left[\%{\text{CD}14}^{+}{\text{cytokine}}^{+}\left(S\right)-\%\text{CD}14^{+}{\text{isotype}}^{+}\left(S\right)\right]-\left[\%{\text{CD}14}^{+}{\text{cytokine}}^{+}\left(U\right)-\text{CD}14^{+}{\text{isotype}}^{+}\left(U\right)\right].$$

(S = stimulated and U = unstimulated cell cultures).

### Data analysis

Statistical tests were performed using graphpad prism version 6.01 for Windows (GraphPad Software, San Diego California USA). A *P* value of <0·05 was considered statistically significant at 95% level of confidence. The Kruskal–Wallis test was used to compare the geometric mean fluorescent intensity (GMFI) values for the markers CD11a, CD11b, CD11c, CD18, TLR‐2, TLR‐4, HLA‐DR and CD86 on monocytes, the percentage of TNF‐α‐ and IL‐6‐producing monocytes and the percentage of haemozoin‐loaded monocytes in different clinical groups. Wilcoxon‐matched pairs test was used to determine the statistical significance of the differences in GMFI values or percentage of cytokine‐producing monocytes observed during acute infection and in convalescence for each clinical type of malaria.

## Results

### Clinical malaria is characterized by low expression of integrins on monocytes

**Figure 2:**
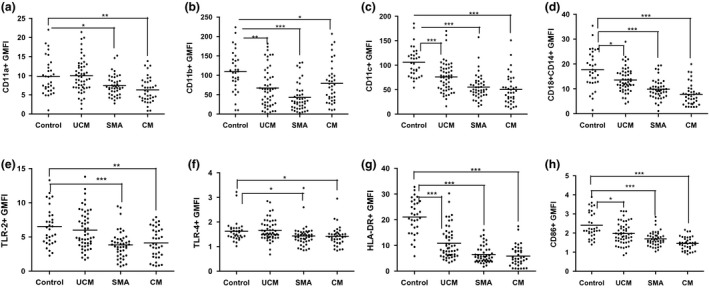
(a to h): Medians (10th and 90th percentiles) of geometric mean florescence intensity (GMFI) of CD11a (a), CD11b (b), CD11c (c), CD18 (d), TLR‐2 (e), TLR‐4 (f), HLA‐DR (g) and CD86 (h) expressions on monocytes from children presenting with UCM, SMA and CM, compared to monocytes from healthy aparasitaemic children. ***P < 0·0001, **P < 0·001, *P < 0·05.

During acute malaria, the mean expression levels of integrins CD11a, CD11b, CD11c and CD18 were significantly lower than in controls for each clinical form of malaria, with the exception of CD11a in UM (Fig. 2a–d). Integrin expression levels significantly recovered in convalescence, except for CD11b expression, which only increased in children with SMA, and CD11c, which decreased in convalescence among children with UM (Fig. 2a–d).

### Clinical malaria is characterized by low expression of TLRs, HLA‐DR and CD86 on monocytes

**Figure 3:**
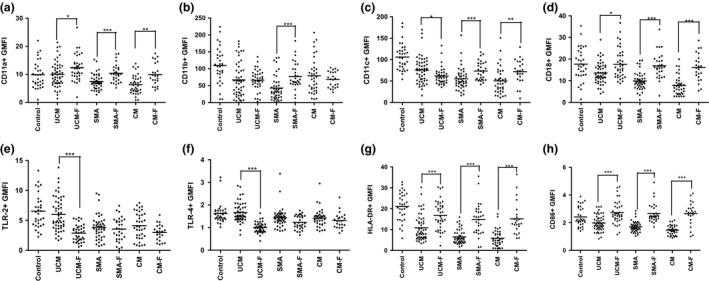
(a to h): Medians (10th and 90th percentiles) of geometric mean florescence intensity (GMFI) of CD11a (a), CD11b (b), CD11c (c), CD18 (d), TLR‐2 (e), TLR‐4 (f), HLA‐DR (g) and CD86 (h) expression on monocytes in children acute malaria (UCM, SMA and CM) and at follow‐up during convalescence (UCM‐F, SMA‐F and CM‐F), compared with monocytes from healthy aparasitaemic children. ***P < 0·0001, **P < 0·001, *P < 0·05.

Mean expression of TLR‐2 and TLR‐4 was significantly lower in children with acute CM and SMA compared with healthy controls (Fig. 2e,f), while HLA‐DR and CD86 expression was significantly lower in all three malaria groups (Fig. 2g,h). In convalescence, the expression of TLR‐2 and TLR‐4 was lower in all three clinical forms of malaria compared to levels observed in acute disease, but this difference was only significant in the UM group. In contrast, expression of CD86 increased significantly in convalescence in all malaria groups and the expression was similar to those observed in healthy controls (Fig. 3h). Although the expression of HLA‐DR in convalescence of all three clinical malaria types was significantly higher than that observed in acute disease, the convalescence expression levels were still lower, though not significantly, than those observed in healthy controls (Fig. 3g).

**Figure 4:**
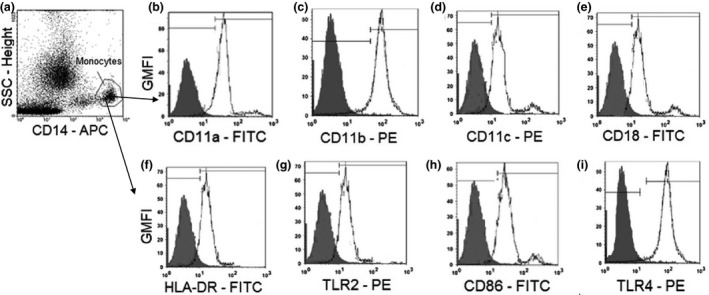
Gating strategy for determining the monocyte population in the side‐scatter/CD14 plot (a), the geometric mean fluorescent intensity (GMFI) values for monocyte surface markers CD11a (b), CD11b (c), CD11c (d), CD18 (e), activation marker HLR‐DR (f), Toll‐like receptor 2 (g), activation marker CD86 (h) and TLR‐4 (i) expressed on monocytes. The grey histograms represent the GMFI of the isotype controls, and the white histogram represents the GMFI of the different surface and activation markers.

### Acute malaria is characterized by a low proportion of TNF‐α‐ and IL‐6‐producing monocytes

**Figure 5:**
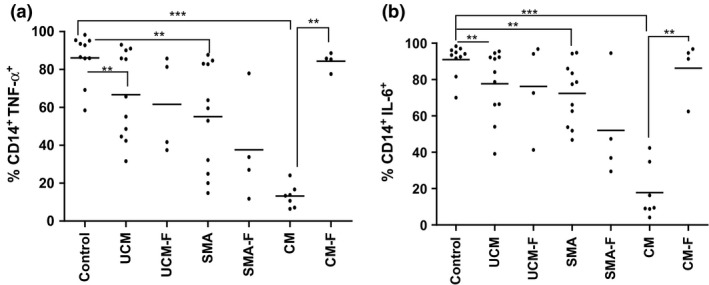
(a to b): Medians (10th and 90th percentiles) of percentage of TNF‐α‐ (a) and IL‐6‐producing monocytes (b) in children presenting with acute disease (UCM, SMA and CM) and at follow‐up during convalescence (UCM‐F, SMA‐F and CM‐F), compared with monocytes from healthy aparasitaemic children. ***<0·0001, **P < 0·001, *P < 0·05.

During acute malaria, percentages of TNF‐α‐ and IL‐6‐producing monocytes in all clinical groups were significantly lower than in controls (Fig. 5a,b), with children with CM having the lowest percentage. In convalescence, there was a marked recovery of TNF‐α‐ and IL‐6‐producing monocytes in children with CM, but for those with SMA, the percentage remained low.

### High percentages of monocytes are loaded with haemozoin in SMA compared with other forms of malaria

In all forms of malaria, haemozoin was visible in a proportion of circulating monocytes by light microscopy (Table S1). Children with SMA had a higher median percentage of haemozoin‐loaded monocytes (17·5%) compared with those with CM (10·0%; *P* = 0·03) and UM (4·0%; *P* < 0·01).

## Discussion

We analysed the expression of different activation and adhesion markers and percentages of cytokine‐producing monocytes in Malawian children presenting with different clinical forms of *P. falciparum* malaria. Expression of most integrins and adhesion markers was reduced significantly during acute malaria especially in children presenting with acute SMA and CM who had the highest proportion of haemozoin‐loaded monocytes, when compared with controls or convalescent levels. Proportions of cytokine‐producing monocytes were also reduced in acute infection of all clinical types of malaria, with the lowest percentages observed for children with CM. Most results normalized in convalescence with the notable exception of pro‐inflammatory cytokine‐producing cells in SMA, and TLR‐2 and TLR‐4 expression during convalescence from all forms of malaria. In all patient groups, a higher proportion of monocytes were loaded with haemozoin than in controls.

Clinical malaria has been characterized by the presence of highly activated circulating T‐cell subsets in a number of studies 5, 28, 29, 30, 31, and we have previously reported the same finding among children in the present study 26. This is consistent with the concept that stimulation of immune effector cells by products from malaria infection results in the release of pro‐inflammatory cytokines responsible for clearing parasites from the circulation 5, 30. Our finding of low expression of integrins, TLRs and activation markers HLA‐DR and CD86, on monocytes during acute malaria, is indicative of a contrasting immunosuppressive effect. However, unlike in the case of TLRs, in which the expression is even lower in convalescence, the expression of the activation markers HLA‐DR and CD86 is higher in convalescence compared to the expression in acute disease, suggesting that the activation markers recover faster from immunosuppressive effect that the integrins.

Ingestion of haemozoin could well be the underlying reason for our observations in monocytes. As mentioned, haemozoin is a product of malaria infection that accumulates in phagocytic cells, including monocytes, and has been implicated in both stimulatory and inhibitory immunological effects 23. These differential effects are likely to be dependent on the time course of exposure to haemozoin and could vary depending on prior malaria exposure and *in vitro* compared with *in vivo* exposure.

Expression of MHC class II (HLA‐DP, HLA‐DQ and HLA‐DR) and CD11c, but not CD11b, has been shown to decrease in monocytes *in vitro* 48 h following phagocytosis of haemozoin 25. In contrast, in another study with a 2 h time course, *in vitro* exposure of leucocytes to isolated malaria pigment from ruptured schizonts resulted in upregulation of CD11b/CD18 on monocytes. The earliest response detected 10 min after exposure to malaria pigment, with a plateau reached at 60 min, followed by a decline at 90 min 32. Both studies were conducted on blood collected from naïve nonexposed adults.

The global low expression of all surface markers examined in the present study, including CD11a, CD11b, CD11c and CD18, could be due to the much longer time course involved. Using samples from children with clinical malaria, monocytes are exposed to malaria‐parasitized red cells and haemozoin for several days prior to the development of clinical symptoms and presentation at hospital. In addition, prior exposure to *P. falciparum* could prime the immunosuppressive effects observed. In contrast, expression of CD11a/CD18 on lymphocytes has been shown to increase in Sudanese children with uncomplicated malaria 33. This could be because, unlike monocytes, lymphocytes do not accumulate haemozoin.

The marked depression of TLR‐2 compared with TLR‐4 expression in acute severe malaria (CM and SMA) could be the result of downregulation of TLR‐2 secondary to binding by *P. falciparum* GPI 14, 34. Low expression of TLR‐2, TLR‐4 and CD86 in acute SMA and CM is consistent with the results of a study 35 assessing the effect of *P. falciparum* macrophage migration inhibitory factor (PfMIF) on monocyte function. This indicates another possible mechanism of monocyte immune suppression to account for our findings. PfMIF is released during malaria and appears to modulate monocyte function, suppressing signalling through TLRs.

In contrast with our findings, Thai adults with severe and mild malaria had raised monocyte TLR‐2 expression, compared with controls, but there was no significant difference in TLR‐4 expression 36. In another study, expression of TLR‐2 or TLR‐4 on monocytes from malaria‐naïve volunteers did not change significantly following 24 h of *in vitro* stimulation with *P. falciparum* lysate 37. In a recent study of *P. falciparum* malaria infection in malaria‐naïve volunteers, monocyte HLA‐DR and CD86 expressions were increased at the time when parasitaemia was detected on thick blood film, prior to drug treatment 38.

All three studies involved clinical material from adults who were naïve to malaria, in contrast to our study among African children with malaria in a *P. falciparum* hyperendemic area. Repeated prior exposure to *P. falciparum* in our age group and setting may account for these reported differences in TLR expression. Immune signalling pathways, including those involving TLRs, are subject to modulation by various mechanisms, most commonly downregulation following stimulation. Stimulation with lipopolysaccharide, the main TLR‐4 ligand, induces tolerance to restimulation 39. Thus, the observation that the expression of both TLR‐2 and TLR‐4 in convalescence of all malaria types was even lower than that observed in acute infection could be a result of such tolerance which could take longer than 30 days to dissipate.

The low frequencies of TNF‐α‐ and IL‐6‐producing monocytes in children with malaria in our study contrast with the presence of activated lymphocyte subsets in malaria with high expression of CD69 26, 28. It is likely that these monocytes were already anergic and refractory to further stimuli when stimulated *in vitro*. Ingestion of haemozoin by monocytes over several days may well have contributed to this refractory state. This is in contrast to enhanced TNF‐α production by monocytes following 1–2 h *in vitro* stimulation with haemozoin 39.

Notably, the fraction of TNF‐α‐ and IL‐6‐producing monocytes in CM was lower than in SMA. One month into convalescence, the percentages in CM children had recovered and were the same as present in controls. In contrast, these percentages were still low in convalescent children with SMA. This sustained suppression of inflammatory cytokine production among children with SMA may contribute to the increased susceptibility of such children to bacteraemia with nontyphoidal strains of *Salmonella* 40. Inflammatory Th1 cytokines, including TNF‐α, are important for protection against nontyphoidal *Salmonella* 41, 42. While the clinical association between invasive nontyphoidal *Salmonella* disease and malaria has been known for a long time 43, this association is most evident in SMA compared with other forms of malaria 44.

Our findings support the concept of immune suppression of monocyte function among African children with malaria, and this may be secondary to phagocytosis of haemozoin. The findings also indicate that monocyte characteristics following exposure to malaria differ depending on the population studied, transmission intensity, prior exposure to *P. falciparum*, patient age and clinical form of malaria. Factors that affect monocyte haemozoin content and length of exposure to haemozoin may account for these differences.

## Author contributions

CAM, WLM, MTD and MEM conceived the study. WLM, CAM, CLM and ENG performed the investigations. WLM, CAM analysed the data. WLM, MEM and CAM wrote the report. CAM oversaw the research. All authors reviewed the report.

## Funding

This work was supported by a PhD studentship from the Gates Malaria Partnership (to WLM) which received support from the Bill and Melinda Gates Foundation, a Wellcome Trust Research Fellowship [grant number 067902/Z/02/Z to C.A.M.], a Wellcome Trust Programme Grant [grant number 074124/Z/04/Z to M.E.M.] and a Clinical Research Fellowship from GlaxoSmithKline to C.A.M.

## Supporting information


**Table S1.** Medians (range) of demographic, clinical and haematological values of participants in first study to characterise expression of monocytes surface markers in different clinical presentations of malaria and in healthy controls.
**Table S2.** Medians (range) of demographic, clinical and haematological values of participants in second to determine the proportion of cytokine‐producing monocytes in different clinical presentations of malaria and in healthy controls.
