# Supplementary material for: Monocyte activation and cytokine production in Malawian children presenting with P. falciparum malaria
Source: Parasite Immunol. 2016 Apr 28;38(5):317–25. doi: 10.1111/pim.12319 (PMC4850749; doi:10.1111/pim.12319)
Supplement: Supplementary file 1 — Table S1. Medians (range) of demographic, clinical and haematological values of participants in first study to characterise expression of monocytes surface markers in different clinical presentations of malaria and in healthy controls. Table S2. Medians (range) of demographic, clinical and haematological values of participants in second to determine the proportion of cytokine‐producing monocytes in different clinical presentations of malaria and in healthy controls. [file PIM-38-317-s001.doc]

**Table S1:** Medians (range) of demographic, clinical and haematological values of participants in first study to characterise expression of monocytes surface markers in different clinical presentations of malaria and in healthy controls.

| **Clinical Group** | **Healthy Controls** | **Uncomplicated**  **Malaria** | **Severe Malarial**  **Anemia** | **Cerebral**  **Malaria** |
| --- | --- | --- | --- | --- |
| **Number** | 42 | 54 | 30 | 29 |
| **Died During Admission** | - | 0 | 1 | 4 |
| **Reviewed in Convalescence** | - | 34 | 21 | 18 |
| **Sex [M:F]** | 29:13 | 38:16 | 19:11 | 10:19 |
| **Age [months]** | 20 (5 - 76) | 27 (6 - 58) | 23 (5 - 38) | 30 (5 - 84) |
| **Parasites/µl of blood** | 0 | 52,300  (460 - 768,000) | 3,500  (20 - 296,000) | 41,800  (900 - 517,000) |
| **Monocyte counts (x 100)/μl**  **Acute** | 0.90 (0.41 – 1.59) | 1.30 (0.60 – 2.87) | 1.50 (0.69 – 3.85) | 0.5 (0.10 – 2.20) |
| **Convalescence** | - | 0.86  (0.52 – 1.80) | 0.80  (0.62 – 1.20) | 0.70  (0.34 – 1.56) |
| **% Haemozoin loaded monocytes** | 0.0  (0.0 – 9.0) | 4.0  (0.0 – 43.0) | 17.5  (0.0 – 62.0) | 10.0  (0.0 – 40.0) |
| **Blantyre Coma Score** | 5  - | 5  - | 5  - | 1  (0-2) |
| **Haemoglobin [g/dL]** | 11.2  (7.0 - 14.1) | 9.3  (5.0 - 13.0) | 3.9  (2.4 - 4.9) | 7.7  (5.3 - 12.5) |

**Table S2:** Medians (range) of demographic, clinical and haematological values of participants in second to determine the proportion of cytokine-producing monocytes in different clinical presentations of malaria and in healthy controls.

| **Clinical Group** | **Healthy Controls** | **Uncomplicated**  **Malaria** | **Severe Malarial**  **Anemia** | **Cerebral**  **Malaria** |
| --- | --- | --- | --- | --- |
| **Number** | 10 | 12 | 12 | 7 |
| **Died During admission** | - | 0 | 0 | 0 |
| **Reviewed in Convalescence** | - | 6 | 4 | 4 |
| **Age [months]** | 18  (12 - 36) | 24  (13 - 60) | 20  (14 - 36) | 32  (18 - 54) |
| **Blantyre Coma Score** | 5  - | 5  - | 5  - | 1  (0 – 2) |
| **Haemoglobin [g/dL]** | 12.6 (8.0 - 15.2) | 10.6 (6.0 - 13.8) | 4.3(2.5 - 4.6) | 8.4 (4.8 - 12.7) |
| **Monocyte counts (x 100)/μl**  **Acute** | 0.85 (0.35 – 1.48) | 1.45 (0.56 – 2.68) | 1.64 (0.72 – 2.98) | 0.45  (0.15 – 2.48) |
| **Convalescence** | - | 0.85  (0.63 – 1.88) | 0.78  (0.56 – 0.98) | 0.71  (0.56 – 0.94) |
